# Supplementary figures and images for: Deterministic Assembly Processes Strengthen the Effects of β-Diversity on Community Biomass of Marine Bacterioplankton
Source: mSystems. 2022 Dec 13;8(1):e00970-22. doi: 10.1128/msystems.00970-22 (PMC9948717; doi:10.1128/msystems.00970-22)

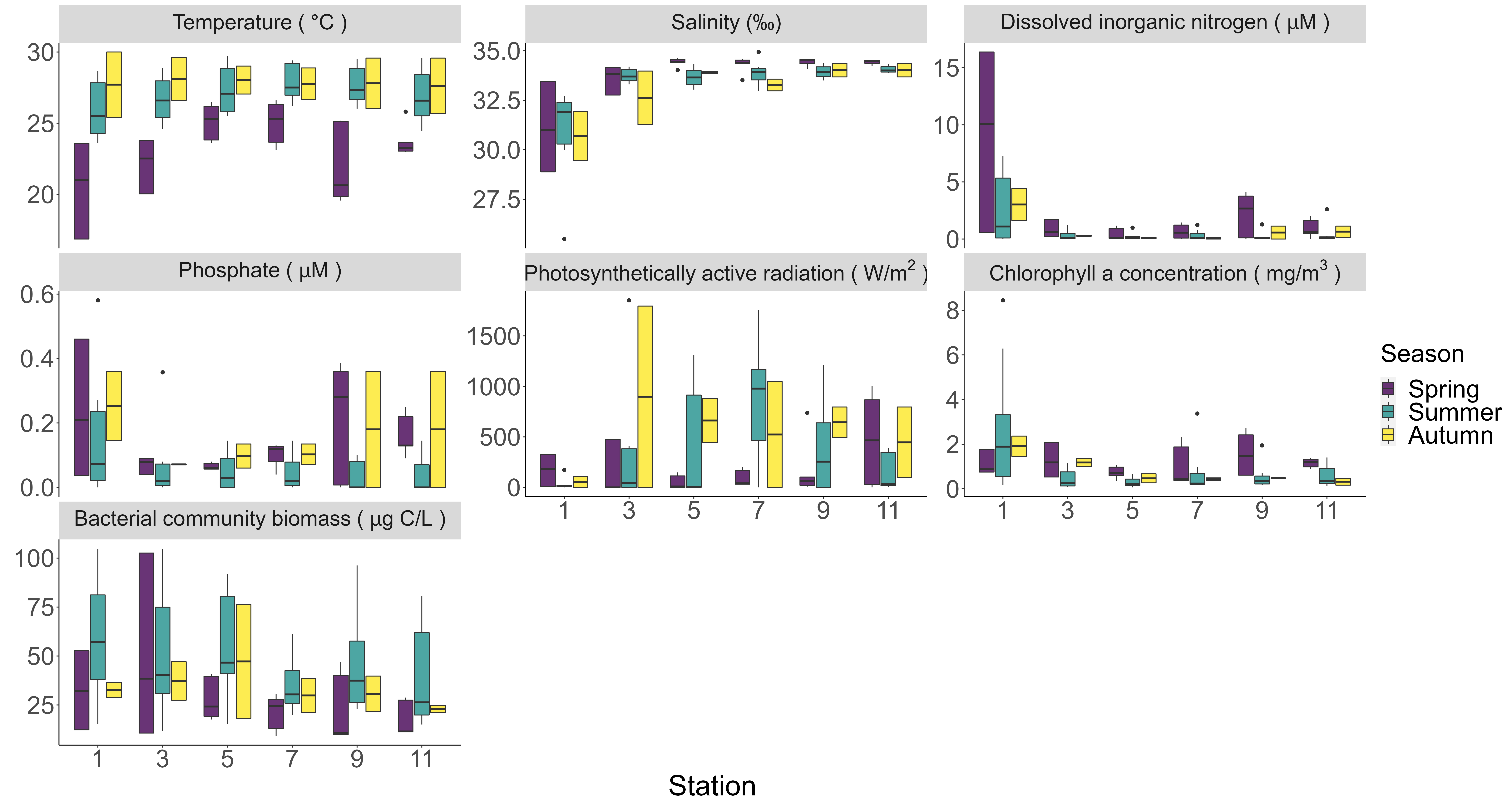

Supplement: FIG S1 [file msystems.00970-22-s0005.tif]

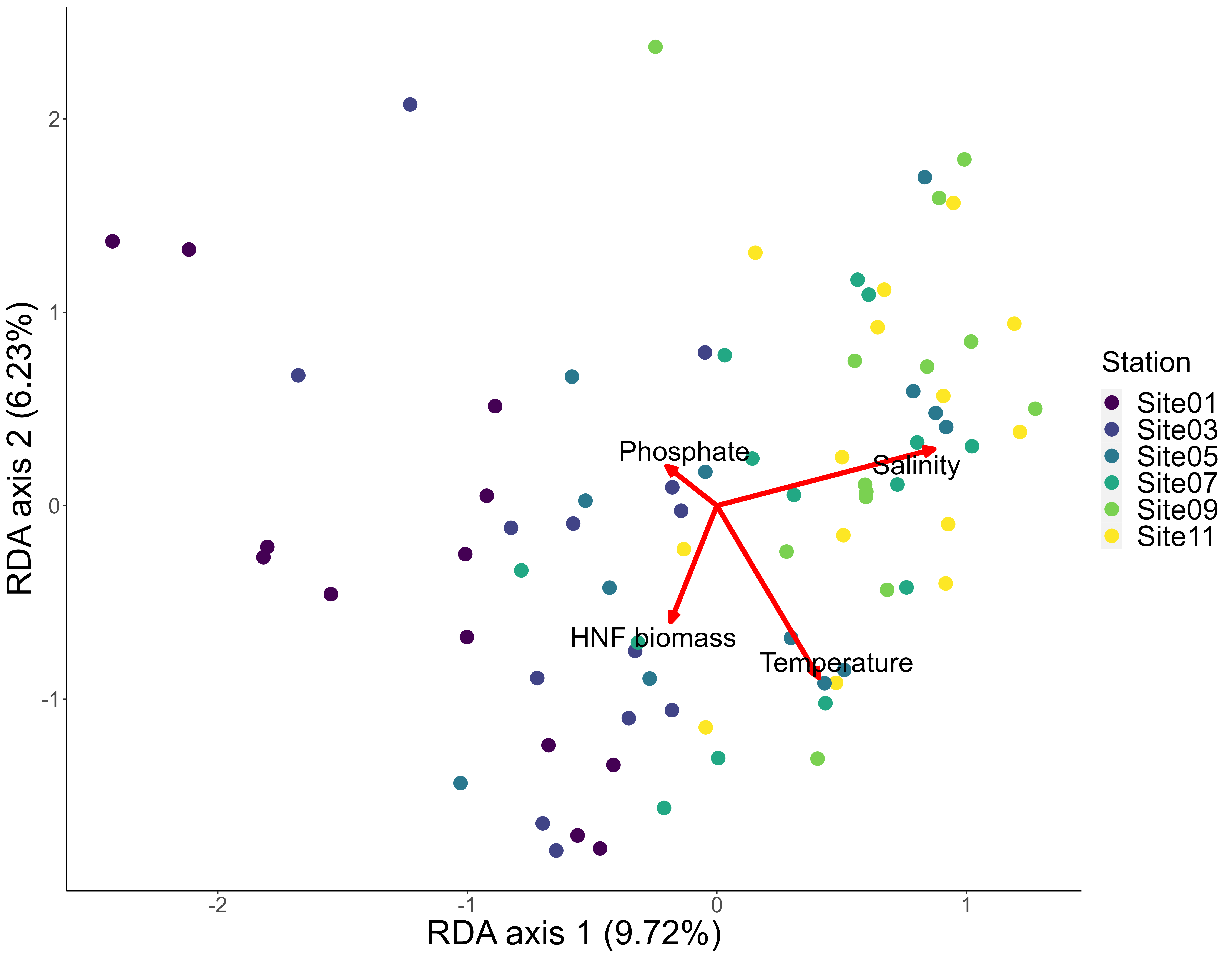

Supplement: FIG S2 [file msystems.00970-22-s0006.tif]

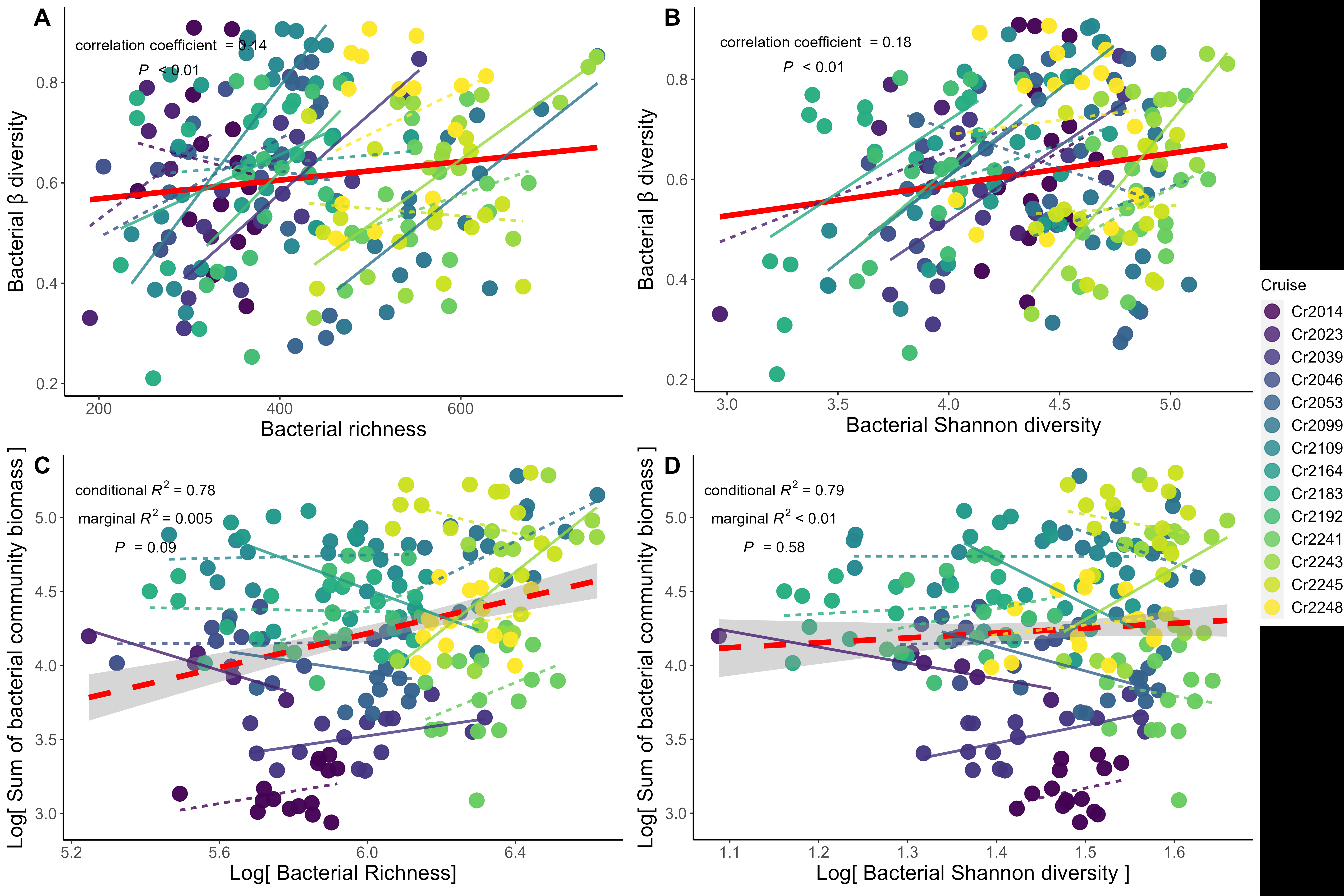

Supplement: FIG S3 [file msystems.00970-22-s0007.tif]

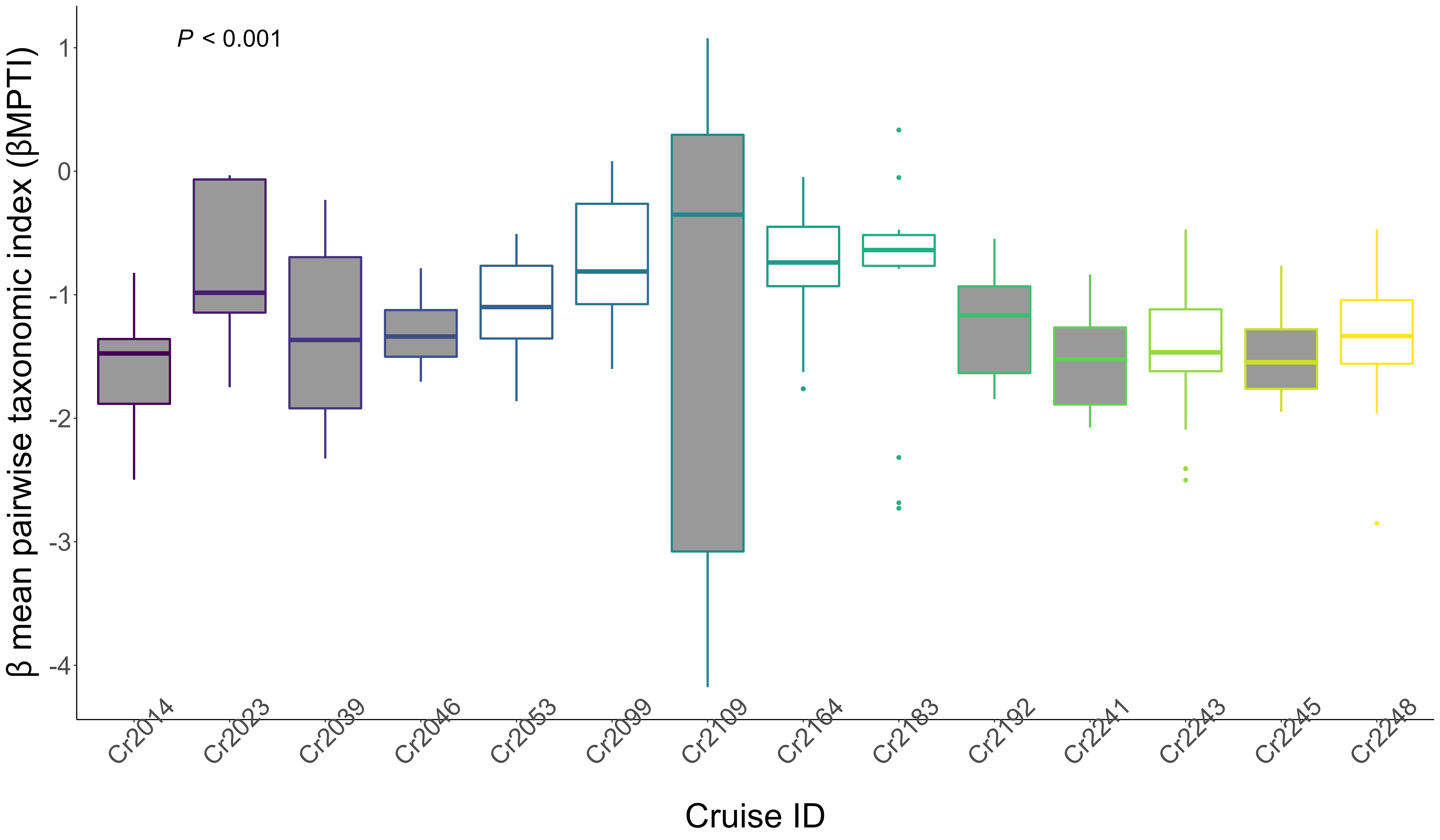

Supplement: FIG S6 [file msystems.00970-22-s0010.tif]

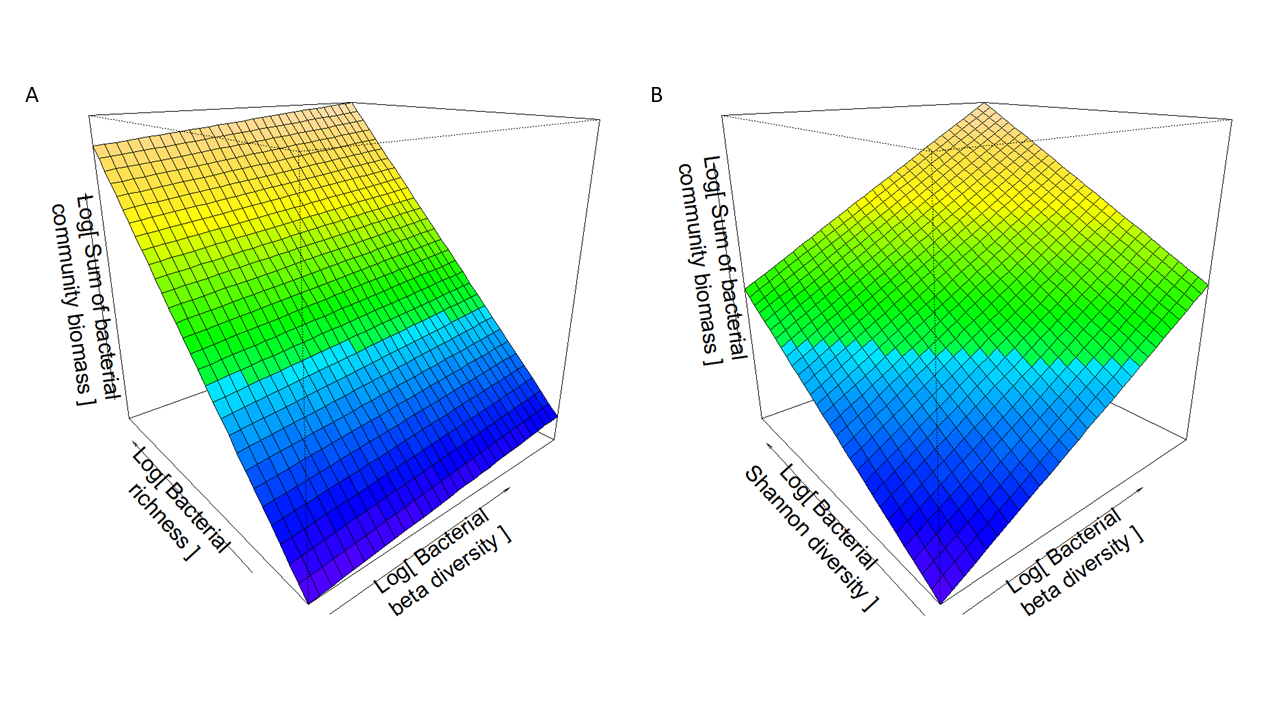

Supplement: FIG S4 [file msystems.00970-22-s0008.tif]

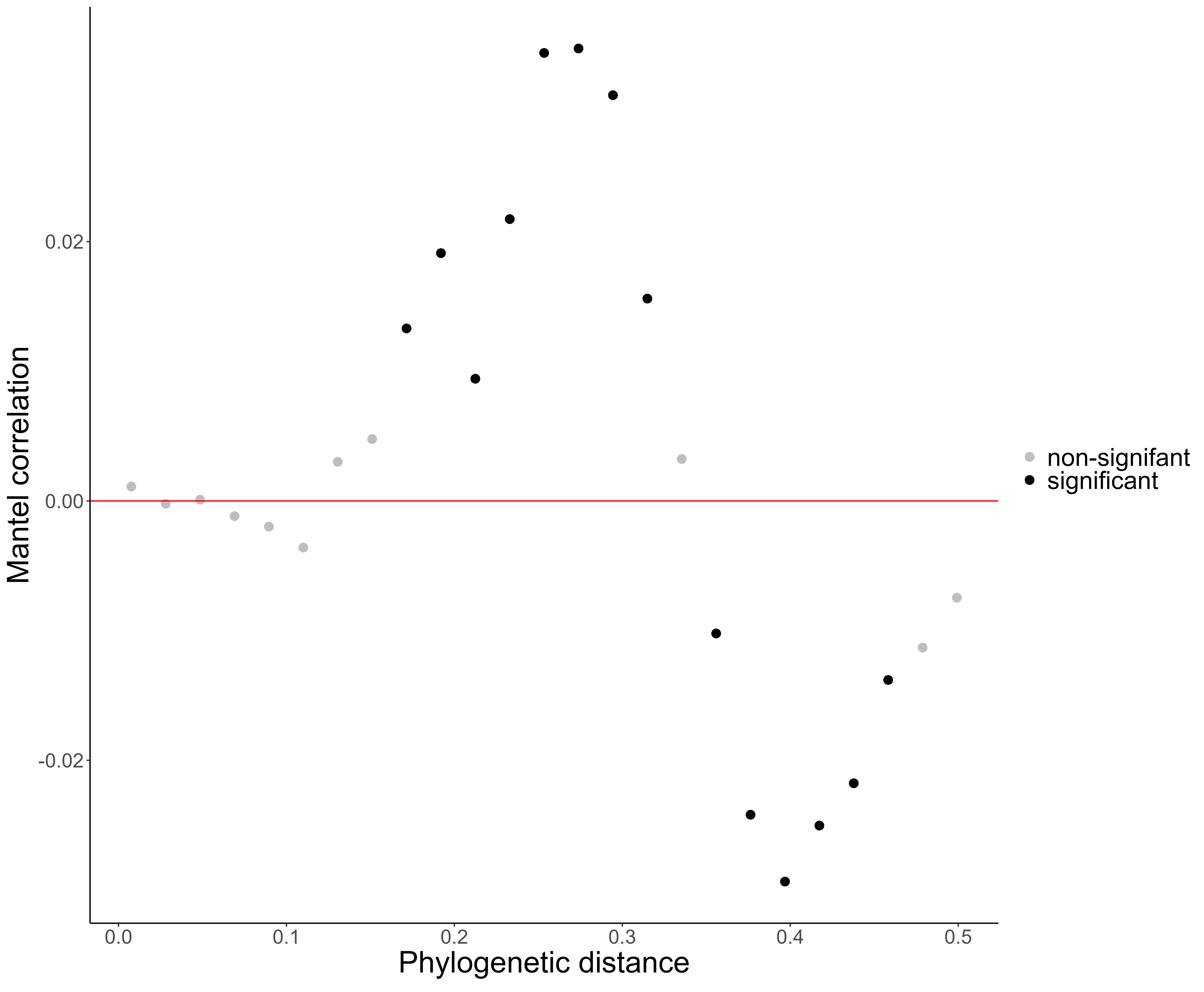

Supplement: FIG S5 [file msystems.00970-22-s0009.tif]
