# Supplementary material for: Deterministic Assembly Processes Strengthen the Effects of β-Diversity on Community Biomass of Marine Bacterioplankton
Source: mSystems. 2022 Dec 13;8(1):e00970-22. doi: 10.1128/msystems.00970-22 (PMC9948717; doi:10.1128/msystems.00970-22)
Supplement: TABLE S2 [file msystems.00970-22-s0003.docx]

Supplementary Table S2.

| Summed bacterial community biomass as the dependent variable | | |
| --- | --- | --- |
|  | Independent variable | p-value |
| Selection step 1 |  |  |
|  | Log (Bacterial β diversity) | 0.02 |
|  | Log (Bacterial Shannon diversity) | 0.63 |
|  | Log (Temperature) | 0.2 |
|  | Log (Salinity) | <0.01 |
|  | Log (Total inorganic nitrogen) | <0.01 |
|  | *Log (Phosphate) | 0.77 |
|  | Log (PAR) | 0.11 |
|  | Log (Chlorophyll-a) | 0.28 |
| Selection step 2 |  |  |
|  | Log (Bacterial β diversity) | 0.02 |
|  | *Log (Bacterial Shannon diversity) | 0.63 |
|  | Log (Temperature) | 0.2 |
|  | Log (Salinity) | <0.01 |
|  | Log (Total inorganic nitrogen) | <0.01 |
|  | Log (PAR) | 0.11 |
|  | Log (Chlorophyll-a) | 0.29 |
| Selection step 3 |  |  |
|  | Log (Bacterial β diversity) | 0.02 |
|  | Log (Temperature) | 0.17 |
|  | Log (Salinity) | <0.01 |
|  | Log (Total inorganic nitrogen) | <0.01 |
|  | Log (PAR) | 0.11 |
|  | *Log (Chlorophyll-a) | 0.33 |
| Selection step 4 |  |  |
|  | Log (Bacterial β diversity) | 0.02 |
|  | *Log (Temperature) | 0.15 |
|  | Log (Salinity) | <0.01 |
|  | Log (Total inorganic nitrogen) | <0.01 |
|  | Log (PAR) | 0.14 |
| Selection step 5 |  |  |
|  | Log (Bacterial β diversity) | 0.03 |
|  | Log (Salinity) | <0.01 |
|  | Log (Total inorganic nitrogen) | <0.01 |
|  | *Log (PAR) | 0.17 |
| Selection step 6 |  |  |
|  | Log (Bacterial β diversity) | 0.04 |
|  | Log (Salinity) | <0.01 |
|  | Log (Total inorganic nitrogen) | <0.01 |
