# Supplementary material for: Deterministic Assembly Processes Strengthen the Effects of β-Diversity on Community Biomass of Marine Bacterioplankton
Source: mSystems. 2022 Dec 13;8(1):e00970-22. doi: 10.1128/msystems.00970-22 (PMC9948717; doi:10.1128/msystems.00970-22)
Supplement: TABLE S3 [file msystems.00970-22-s0004.docx]

Supplementary Table S3.

| Bacterial β diversity effects on the summed bacterial community biomass as the dependent variable | | |
| --- | --- | --- |
|  | Independent variable | p-value |
| Selection step 1 |  |  |
|  | $\beta MPTI$ | <0.01 |
|  | *Log (Temperature) | 0.22 |
|  | Log (Salinity) | <0.01 |
|  | Log (Total inorganic nitrogen) | <0.01 |
|  | Log (Phosphate) | <0.01 |
|  | Log (PAR) | 0.01 |
|  | Log (Chlorophyll-a concentration) | <0.01 |
| Selection step 2 |  |  |
|  | $\beta MPTI$ | <0.01 |
|  | Log (Salinity) | <0.01 |
|  | Log (Total inorganic nitrogen) | <0.01 |
|  | Log (Phosphate) | <0.01 |
|  | Log (PAR) | 0.01 |
|  | Log (Chlorophyll-a) | <0.01 |
