# Supplementary material for: Deterministic Assembly Processes Strengthen the Effects of β-Diversity on Community Biomass of Marine Bacterioplankton
Source: mSystems. 2022 Dec 13;8(1):e00970-22. doi: 10.1128/msystems.00970-22 (PMC9948717; doi:10.1128/msystems.00970-22)
Supplement: TEXT S1 [file msystems.00970-22-s0001.docx]

**Supplement I:**

1.1 Library preparation and sequencing

Total DNA was extracted separately from the 0.2 μm-pore size filters with the PowerWater DNA Extraction Kit (Qiagen) according to the manufacturer’s instructions. DNA extracts from the 0.2-μm-pore size filters were used as templates of polymerase chain reaction (PCR) for 16S sequencing. PCR was performed in two steps: to amplify targeted 16S regions in the first step PCR, and attach sample-specific barcodes for each sample in the second step PCR [1]⁠.

For the prokaryotic 16S rDNA, the V5–V6 region of 16S rDNA was amplified using the forward primer FIA-787F (5’-[illumina forward index adaptor]-ATTAGATACCCNGGTAG-3’) and reverse primer RIA-1046R (5’-[illumina reverse index adaptor]-CGACAGCCATGCANCACCT-3’) [2]⁠. In the first step PCR, 20-μl of PCR mix contained: 1 U of Taq DNA polymerase (Promega), 1 × reaction buffer, 1.5 mM MgCl2, 0.2 mM dNTPs, 0.2 mM of primers and 2 ng DNA templates. Conditions for the PCR cycles were: an initial denaturation at 94 °C for 3 min; 25 cycles of 94 °C for 30 s, 55 °C for 45 s, 72 °C for 1 min; and a final extension at 72 °C for 2 min. Three PCR replicates were conducted and pooled for each sample for obtaining enough DNA concentration for sequencing.

DNA products of the first step PCR were purified using AMPure XP beads (Beckman Coulter Genomic, CA, USA) and DNA concentration was quantified using a Qubit fluorometer (Invitrogen, Carlsbad, CA, USA) with Qubit dsDNA BR Assay Kit (Life Technologies, USA) before conducting the second step PCR.

Primers containing sample-specific barcodes and Illumina adaptors were used to perform the second step PCR. 20-μl of PCR mix contained: 1 U of Taq DNA polymerase (Promega), 1 × reaction buffer, 1.5 mM MgCl2, 0.2 mM dNTPs, 0.2 mM of S5, and N7 primers (Nextera Index Kit) and 2 ng DNA obtained from the first step PCR. The PCR conditions was with initial denaturation at 94 °C for 3 min; 6 cycles of 94 °C for 30 s, 55 °C for 45 s, 72 °C for 1 min; and a final extension at 72 °C for 2 min. For this, three PCR replicates were conducted and pooled for each sample. Finally, DNA samples with unique barcodes were pooled in roughly equal concentrations and sent for 2 × 300 bp paired-end sequencing with Illumina Miseq platform. The sequence data have been deposited in the NCBI Sequence Read Archive (SRA) under the accession numbers: PRJNA662424.

1.2 Sequence processing

Sequences were analyzed using DADA2 v.1.12 [3]⁠ pipeline running on R v.3.6.2. Suggested by DADA2 pipeline tutorial, paired-end sequences were filtered and trimmed separately depends on sequences quality by using function “filterANDTrim” before DADA2 algorism. Primer sequences were trimmed, and sequences were truncated at position 220-bp (for forward reads) and 180-bp (for reverse reads) for 16S rDNA sequences, where average quality score start to crash lower than Q30. We thus include the sequences that have average quality score greater than Q30. Sequences contain Ns and more than two expected errors were also removed. DADA2 algorithm was then performed to remove spurious sequences that possibly be generated during PCR amplification and sequencing [3]⁠. After quality filtration, paired-end reads were merged using function “mergePairs” with a minimum of 12-bp overlap. Merged sequences were then assembled in to ASVs (amplicon sequence variant) with function “makeSequenceTable”. Finally, chimeras were detected and removed from ASV pool using “removeBimeraDenovo”.

Taxonomy assignment was preformed to recognize bacterial ASVs from 16S rDNA. Six taxonomy levels (from phylum to the genus) were assigned using Naïve Bayesian Classifier [4]⁠ implemented in function “assignTaxonomy”. The exact matching to the species level was assigned using function “assignSpecies”. For reference databases, Silva 132 database [5]⁠ was used for assigning 16S rDNA reads. Subsequently, bacterial communities were selected as ASVs classified under kingdom “bacteria”.

With the aim to quantify bacteria community assembly processes with phylogeny information, phylogenetic trees were built with maximum likelihood method using the “phangorn” package in R (with negative edges length = 0).

1.3 Phylogenetic signal calculation

We show the phylogenetic signal for the bacteria communities in the southern East China Sea. To do so, we first quantified the abiotic habitats for each ASV (amplicon sequence variant) by calculating the abundance-weighted average of environmental variables (including temperature, salinity, photosynthetic active radiation, nitrite, nitrate and phosphate concentrations). We then plotted the Mantel correlations between ASVs’ habitats against the phylogenetic distance among all ASVs (Figure S6). The phylogenetic signal was detected using the mantel.correlog function in the vegan package in R4.0.0. Significant phylogenetic signal suggests that phylogenetic distances can be used to approximate the ecological niche of species [6]

**Supplementary references**

1. Berry D, Mahfoudh BK, Wagner M, Loy A. Barcoded primers used in multiplex amplicon pyrosequencing bias amplification. Appl Environ Microbiol 2011; 77: 7846–7849.

2. Cai L, Ye L, Tong AH, Lok S, Zhang T. Biased diversity metrics revealed by bacterial 16S pyrotags derived from different primer sets. PLoS One 2013; 8: e53649.

3. Callahan BJ, McMurdie PJ, Rosen MJ, Han AW, Johnson AJA, Holmes SP. DADA2: high-resolution sample inference from Illumina amplicon data. Nat Methods 2016; 13: 581–583.

4. Wang Y, Liu J, Zhao H, Lü W, Zhao J, Yang L, et al. Human 1A6/DRIM, the homolog of yeast Utp20, functions in the 18S rRNA processing. Biochim Biophys Acta 2007; 1773: 863–868.

5. Quast C, Pruesse E, Yilmaz P, Gerken J, Schweer T, Yarza P, et al. The SILVA ribosomal RNA gene database project: improved data processing and web-based tools. Nucleic Acids Res 2013; 41: D590–D596.

6. Losos JB. Phylogenetic niche conservatism, phylogenetic signal and the relationship between phylogenetic relatedness and ecological similarity among species. Ecol Lett 2008; 11: 995–1003.
